# Influence of Lifestyle and Environmental Factors on Semen Quality in Ghanaian Men

**DOI:** 10.1155/2020/6908458

**Published:** 2020-10-21

**Authors:** Richard Michael Blay, Abigail Duah Pinamang, Augustine E. Sagoe, Ewurama Dedea Ampadu Owusu, Nii Koney-Kwaku Koney, Benjamin Arko-Boham

**Affiliations:** ^1^Department of Anatomy, University of Ghana Medical School, College of Health Sciences, University of Ghana, Korle Bu, Accra, Ghana; ^2^Department of Medical Laboratory Sciences, School of Biomedical and Allied Health Sciences, College of Health Sciences, University of Ghana, Korle Bu, Accra, Ghana; ^3^Central Medical Laboratory, Immunology Unit, Korle-Bu Teaching Hospital, Korle Bu, Accra, Ghana

## Abstract

**Introduction:**

Male infertility is known to contribute about half of all infertility cases. In Ghana, the prevalence of male infertility is higher (15.8%) than in females (11.8%). Sperm quality is associated with the likelihood of pregnancy and known to be the cause of male fertility problems 90% of the time. Exposure to certain environmental factors reduces semen quality in men. The study examined the effects of environmental and lifestyle factors on semen quality in Ghanaian men.

**Materials and Methods:**

This was a cross-sectional study involving 80 apparent healthy adult males in their reproductive age. Participants were males referred to the laboratory (Immunology Unit of the Korle-Bu Teaching Hospital) for semen analysis test and/or culture and sensitivity. Participants were made to fill out a questionnaire which entailed selected environmental factors (accidents or trauma, exposure to chemicals, radiation, and heat) and lifestyle habits (including alcohol consumption, smoking, and whether participants sat more or less than 4 hours per day). Semen samples were then collected by masturbation into sterile containers and analysed in accordance with WHO guidance for semen analysis within 60 minutes after ejaculation and collection.

**Results:**

About 69% of participants had semen pH within the normal range compared to 15% whose pH were lower than 7.2. There was a significantly high number of immotile sperm cells (*p* value = 0.017) in participants who sat for more than 4 hours as compared to those that sat for less than 4 hours in a day. Active sperm motility and viability showed significant increase (*p* value = 0.002 and 0.009, respectively) in participants who kept their cell phones in their side pockets. Smoking produced a twofold decrease in sperm count as smokers had a significantly lower sperm count (12.28 ± 10.95 × 10^6^/ml) compared to the smoke-free (23.85 ± 22.14 × 10^6^/ml). For exposure to STDs, no significant differences were recorded among study groups concerning semen quality.

**Conclusion:**

Sperm quality in Ghanaian men is associated with lifestyle habits. Smoking and sitting for long hours influenced sperm motility and count, respectively. Knowledge of the factors that influence sperm quality in this geographical region can contribute to informed decisions on effective management of infertility in Ghanaian men.

## 1. Introduction

Male infertility is known to contribute to about half of all infertility cases and is usually the most difficult form to treat [1]. The prevalence of infertility may be higher in males than in females, indicating the importance of studying the male contribution to infertility [2]. Sperm count and concentration are strongly associated with the likelihood of pregnancy and known to be the cause of male fertility problems 90% of the time [3]. The rapid decline in global fertility rates in recent decades has occurred alongside decreasing sperm count and quality suggesting that decline in fertility may be due to decreasing sperm quality [4–6]. Meta-analysis studies show that there has been a 57% reduction in sperm concentration around the globe over the past 35 years, 32.5% decline over the past 50 years in Europe, and a 72.6% decline found in Africa over the past 50 years [7]. Although several factors may affect male fertility and sperm quality, industrialization and economic growth have been shown to be a significant contributor due to increased exposure to pollutants, changes in diet, and lifestyle [8, 9]. Recent studies show that exposure to certain environmental factors such as noise pollution and lifestyle habits such as sitting for more than 6 hours per day, tobacco use, alcohol, testicular heat stress, radiation, and obesity all reduce semen quality in men [10, 11]. A systematic review [9] of literature in association with lifestyle factors and male infertility over the last 10 years using PubMed showed that smoking, alcohol, obesity, diet, psychological stress, advanced paternal age, genital heat stress, and sleep disturbances may all adversely impact sperm quality. Another report [12] also showed evidence that diet, extreme physical activity, and body mass index (BMI) may also have adverse effects on male fertility. Furthermore, there is evidence that low sperm quality, occupational exposure, and unhealthy lifestyle habits in men may be associated with recurrent pregnancy loss (RPL) in their partners [13] suggesting far-reaching effect of environmental exposure and lifestyle on the reproductive health of men. Work and life stress factors that are perceived to be high in modern populations are also associated with sperm concentration, motility, and morphology [14]. Yang et al. [15] reported that smoking and consumption of caffeinated drinks and fried foods were significantly associated with poor semen quality. Other studies have also suggested that prolonged use of mobile phones and the wearing of tight underwear also impact negatively on semen parameters and DNA integrity [16].

The mechanism by which lifestyle and environmental factors reduce the quality of sperms is not fully understood; however, there are studies that suggest disruption of endocrine function, aberrant DNA methylation in sperm cells, abnormal seminal plasma zinc levels, and morphological damage to sperms [17–21]. A recent publication has shown several genes linked to fertility, and mutations to these single genes lead to low sperm quality [22]. To understand decreasing fertility rates and factors that lead to genetic and physiological aberrations resulting in low sperm quality, this study examined the influence of environmental and lifestyle factors on parameters of semen quality in Ghanaian men.

## 2. Materials and Methods

The study was approved by the Ethics and Protocol Review Committee of the School of Biomedical and Allied Health Sciences, College of Health Sciences, University of Ghana (Ethics Identification Number: SBAHS-MD./10468875/AA/5A/2016-2017). Informed consent was obtained from participants prior to study enrolment.

### 2.1. Study Participants

This was a cross-sectional study conducted at the Immunology Unit of the Korle-Bu Teaching Hospital (KBTH), Accra. The study participants were adult males in their reproductive years referred to the Immunology Unit of the Central Laboratory of the KBTH for semen analysis. To be eligible, a participant should have been referred to the Unit by a qualified medical officer. These are males seeking medical attention for difficulties with conception with their spouses. In addition, eligible participants should not have ejaculated at least 3 days prior to the day of investigation. All those who did not meet these criteria were excluded from the study.

### 2.2. Data Collection on Lifestyle Factors

A structured questionnaire which had previously been pretested was administered to consented and enrolled participants. Participants responded to close-ended questions bordering on their lifestyle such as smoking habit, alcohol intake (daily, weekly, or occasionally), the type of underwear, approximate number of sitting hours per day including occupation and the use of cell phones and the site of common storage on the body, and environmental factors, including exposure to X-ray and participants' history on sexually transmitted diseases (STDs). Participants were classified as smokers (exposed) if they indicated they did smoke either daily or occasionally and nonsmokers (unexposed) if they never smoked.

The face validity approach was used to validate the questionnaire. In doing that, the structured questionnaire was first reviewed by an expert in male reproductive health, after which the questionnaire was piloted in a selected population.

### 2.3. Sample Collection

Participants were asked to fill out a questionnaire which entailed selected environmental factors and lifestyle habits. Selected environmental factors included accidents or trauma, exposure to chemicals, radiation, and heat. Lifestyle habits investigated included number of hours spent sitting, alcohol consumption, and smoking. Semen samples were then provided by participants using masturbation method into sterile containers for analysis.

### 2.4. Semen Analysis

Semen samples were analysed in accordance with World Health Organisation (WHO) guidelines and reference values [23] for semen analysis within 60 minutes after ejaculation. Macroscopic examination was performed to determine parameters such as pH, volume, colour of semen, liquefaction, and consistency. Microscopic examination was also performed to determine sperm count, sperm morphology, and sperm motility.

### 2.5. Macroscopic Examination

Semen appearance, using colour, was noted, and the volume was measured using a graduated Pasteur pipette. The semen was then allowed to drop from the Pasteur pipette by gravity, and the length of the thread was observed for liquefaction. Semen pH was determined using a pH strip. According to the WHO Manual [23], normal ranges for volume and pH are 1.5 ml-5 ml and 7.2-8.0, respectively.

### 2.6. Microscopic Examination

To assess sperm motility, a drop of semen was delivered onto a treated clean glass slide and covered with a 22 × 22 mm coverslip. The slide was examined under a light microscope using objective lens of ×40, to count a total of 100 spermatozoa. The motility of the sperms was graded as active progression, sluggish progression, and immotile. Active progression meant spermatozoa moved actively, either linearly or in a large circle. Sluggish progression referred to “all other patterns of motility with an absence of progression, e.g. progressing in small circles, the flagellar force hardly displacing the head, or when only a flagellar beat can be observed” [23]. The percentage of active, sluggish, and immotile spermatozoa was assessed for each sample. According to the WHO guidelines, for semen sample to be considered having normal motility, >50% must have active progression, <20% must have sluggish progression, and <30% must be immotile.

### 2.7. Sperm Viability

Sperm viability is the live sperm cells independent of their motility. A drop of semen was mixed with a drop of 0.5% eosin Y stain in NaCl (Labtech Chemicals) at room temperature, and a smear was prepared from the mixture and observed under a light microscope using ×40 objective lens. A total of 100 spermatozoa was counted, and the percentage of live sperm cells estimated. A viability >70% was considered normal. The nonviable sperm cells pick the eosin stain because their cell walls are not intact.

### 2.8. Sperm Morphology

A thin smear of well-mixed semen was prepared on a slide and fixed in 95% ethanol whiles smear was still wet for 5-10 minutes. It was then allowed to air-dry. The smear was washed with a sodium bicarbonate-formalin solution to remove any mucus present. It was then rinsed in different changes of water. The smear was covered with dilute (1 in 20) carbol fuchsin (Blulux Laboratories Limited) and allowed to stain for 3 minutes. The stain was washed off with water. A counter stain of dilute (1 in 20) methylene blue (Avondale Laboratories Limited) was used to cover the smear for 2 minutes and washed off with water. The smear was allowed to air-dry and observed with a microscope at ×100 using oil immersion.

### 2.9. Sperm Count

Using a haemocytometer, a 1 in 20 dilution from each well-mixed sample was prepared by diluting 50 *μ*l of liquefied semen in 950 *μ*l of sodium bicarbonate-formalin solution. The Neubauer chamber was charged with the well-mixed solution, and the spermatozoa were allowed to settle within 3-5 minutes. Using ×10 objective lens, sperm cells in an area of 2 mm^2^ were counted. The total number of sperm cells counted were multiplied by 1 × 10^5^ to calculate total sperm count. A sperm count less than 20 million and more than 20 million was recorded as oligospermia and normospermia, respectively. Semen with no sperm cells was recorded as azoospermia.

### 2.10. Data Analyses

Data were analysed using Microsoft Excel 2010 and IBM Statistical Package for Social Sciences (SPSS) version 20.0. Independent Student *t*-test was used to analyse continuous variables and presented as tabulated means and standard deviations. Categorical variables were presented as frequencies and percentages and analysed using Pearson's chi-squared test, with Yates' or Fisher's continuity correction when necessary.

## 3. Results

### 3.1. Demography of Study Participants

Eighty (80) men between the ages of 21 and 62 years were recruited into the study. The mean age of participants was 39 years. As shown in Table 1, the largest age group was 31–40 years comprising 48.75%, whereas the least age group represented was above 50 years of age (5%).

### 3.2. Semen Parameters among Participants

The variables measured in the semen analyses including volume, pH, motility, morphology, viability, and count are shown in Table 2. With reference to the WHO manual for examination and processing of human semen, 72.5% of the samples were within the normal range for volume, whereas 22.5% had semen ejaculate volume below the lower reference limit of 1.5 ml. About 69% of participants had semen pH within the normal range compared to 15% whose pH were lower than 7.2. Motility, viability, and sperm count are also indicated in Table 2.

### 3.3. Lifestyle Habits and Semen Quality

#### 3.3.1. Effect of Long Hours of Sitting on Semen Quality

Among the participants, 61.25% reported that they sit for 1-4 hours per day whiles 38.75% reported sitting for more than 4 hours per day. The number of hours spent sitting involved hours spent at work and in sedentary living. Participants were grouped into two categories, ≤4 hours and >4 hours as shown in Table 3. There was a significantly high number of immotile sperm cells (*p* value = 0.017) in participants who sat for more than 4 hours as compared to those that sat for less than 4 hours in a day. Other semen parameters like sperm count, viability, and volume showed no significant differences in relation to the average number of hours participants sat per day.

### 3.4. Effect of Alcohol Consumption and Smoking on Semen Quality

About 8.8% of the study participants were smokers, and 35% indicated they consumed alcohol. Participants were grouped into two categories, exposed (yes) and unexposed (no). Table 3 shows the means and standard deviations of participants who consume alcohol. There were no statistical differences between semen parameters of those that consumed alcohol and those who did not.

Table 3 also shows the means and standard deviations of semen parameters of participants who smoked and those that did not. Apart from a significant decrease in sperm count (*p* value = 0.036), there were no statistical differences between other semen parameters of smokers and nonsmokers.

### 3.5. Environmental Factors and Semen Quality

The effects of environmental factors such as exposure to X-ray, STDs, and site of mobile phone storage were assessed. Previous exposure to X-ray was reported by 11.25% of participants, and 25% had contracted a sexually transmitted disease (STD) within the last 2 years.

### 3.6. Site of Mobile Phone Storage and Semen Quality

Mobile phone storage sites were grouped into two categories, side pockets and other places (including breast pockets and hand bags). Among the participants recruited in this study, 15% commonly kept their mobile phones in other places such as back pockets and breast pockets and 85% commonly kept their mobile phones in their side pocket which is also closer to the groin. As shown in Table 4, active motility showed significant increase with a *p* value of 0.002 in participants who kept phones in their side pockets. Viability also showed a significant increase with a *p* value of 0.009 in participants who kept their mobile phones in their side pockets. Other parameters from semen analysis did not show any significant increase or decrease.

### 3.7. Exposure to X-Rays and Semen Quality

Table 4 shows the means and standard deviation of X-ray exposure. Participants were grouped into two categories, exposed (yes) and unexposed (no). Immotile sperm cells showed significant decrease with a *p* value of 0.000 in participant who were exposed to X-ray. A significant increase was also shown in viability of participants who were exposed to X-ray with a *p* value of 0.036. Other parameters from semen analysis did not show any significant increase or decrease.

### 3.8. Exposure of Participants to STDs and Semen Quality

Table 4 shows the means and standard deviation of participants who have had STDs. Participants were grouped into two categories, exposed (yes) and unexposed (no). No significant increase or decrease was recorded in the semen quality.

## 4. Discussion

Decline in sperm quality around the globe has necessitated the need to understand how modern lifestyle patterns and exposure to environmental hazards have contributed to this phenomenon. We examined the effect of exposure to factors such as X-rays, STDs, and mobile phone usage and storage on sperm quality, as well as the effect of lifestyle habits like long sitting hours, smoking, and alcohol consumption. Sperm quality was assessed by measuring volume of ejaculate, pH, motility, viability, and sperm count. Smoking and sitting for more than 4 hours in a day were associated with decreased sperm count and high number of immotile sperms, respectively. Semen parameters of participants who consumed alcohol were not significantly different from those that did not. Exposure to X-rays and site of mobile phone storage were found to affect semen parameters whiles exposure to sexually transmitted diseases had no effect on semen parameters in participants.

In this study, volume and pH of semen were neither influenced by exposure to any of the environmental hazards nor lifestyle practices assessed. This may be because the hazards included in our study were not of chemical nature. Exposure to environmental chemicals has been shown to increase the concentration of the chemical components in semen, suggesting that environmental factors can affect the biochemical composition of semen [24]. Although semen volume and pH are known to affect motility [25], results of the current study did not show any environmental factor that significantly changed the pH or volume of semen.

Our study also shows that sitting for more than 4 hours a day and smoking may affect sperm motility and count, respectively. Men who reported to sit for more than 4 hours a day had significantly high number of immotile sperms, and the number of active sperms was also lower compared with men who sat for less than 4 hours in a day. This study used the length of hours participants reported to sit per day as a proxy for physical activity. Although the results showed no effect of long sitting hours on sperm count and viability, participants that sat for longer hours had a higher percentage of their sperms scored as immotile and this finding is similar to a previous study conducted by Jóźków et al. [26]. Sitting for long hours may lead to increased scrotal temperature and impairment of spermatogenesis [27] and hence reduced motility. Sitting for more than 4 hours in this study may indicate a sedentary lifestyle or inadequate physical activity. According to Lalinde-Acevedo et al. [28], physical activity improves semen qualities and this may be due to the fact that physically active men have lower fat mass compared to inactive men [29]. Increased fat mass in men reduces plasma testosterone levels and also increases gonadal temperatures due to increased fat tissue around the testes [30]. Furthermore, it is reported that resistance exercises may reduce inflammation and oxidative stress in physically active men, thereby improving semen quality [31]. Studies on exercises and sperm quality have shown inconsistent results, and as such, more reliable and reproducible experiments will have to be designed [32, 33].

We examined the influence of cigarette smoking on semen quality owing to the fact that smoking has been associated with several pathological changes, and retrospective studies have shown smoking to lower semen volume, sperm count, and motility [34–36]. Our results showed that men who reported to smoke had a twofold decrease in sperm count. Smoking may reduce sperm quality due to the effect of nicotine and reduced concentration of zinc in semen [37]. In 2016, a study by Asare-Anane et al. [38] showed that smoking reduced semen volume, pH, viability, morphology, and sperm count, with the effects on count, viability, and morphology more pronounced in men who smoked more sticks per day. They suggested that smoking may decrease sperm quality by reducing testosterone levels in smokers. Put together, the data suggests that cigarette smoking reduces sperm quality through multiple pathways including reduced testosterone levels and zinc concentration in semen.

Our study found no significant differences in sperm parameters of participants who consumed alcohol and those that did not. This is in contrast to previous studies that reported that habitual consumption of alcohol reduced sperm quality [39, 40]. On the other hand, a systematic review of studies on alcohol intake and semen quality showed that moderate drinking did not affect semen parameters, but daily consumption affected semen quality [40]. Our results did not show significant differences in semen parameters mainly due to the fact that our data compares occasional consumption versus no consumption [40]. Whereas the study categorized responses into exposure to alcohol and nonexposure, more reliable results may be obtained by differentiating between moderate and daily or high levels of alcohol intake. Nevertheless, the present data shows that occasional alcohol consumption may not negatively impact semen quality.

We also investigated the effect of mobile phone usage and exposure to mobile phone radiations on semen and sperm quality by taking into consideration the site of common storage of the mobile phones on the body. The side pockets were thought to be closest to the groin and therefore may expose the testes and other related structures to the greatest radiation insult, but on the contrary, our study showed phone storage in the side pocket may increase sperm motility and viability. Previous studies have reported reduced motility and morphology of sperms due to radiation, thermal effect, and increased oxidative stress caused by the use of mobile phones [41]. The contradictory results may be due to huge disparity in the percentage of participants that stored mobile phones in side pockets (85%) compared to those that reported to store mobile phones in the breast pocket and other places like in handbags. Moreover, our study did not take into account the type of mobile device as well as the length of time the participants had been using the device. Future studies must take into account these factors. Another limitation of the study may be that semen samples were collected from men referred to the laboratory indicating possible underlying fertility issues as well as the fact that semen collection was done once as opposed to possibly collecting multiple samples for analysis.

## 5. Conclusions

Sperm quality in Ghanaian men is associated with lifestyle habits. Smoking and sitting for long hours in a day influenced sperm motility and count, respectively. Knowledge of the factors that influence sperm quality in this geographical region can contribute to informed decisions on effective management of infertility in Ghanaian men.

## Figures and Tables

**Table 1:** Age distribution of participants.

Age group	Frequency	Percentage (%)
21-30	15	18.75
31-40	39	48.75
41-50	22	27.5
>50	4	5
Total	80	100

**Table 2:** Mean values of semen parameters of participants.

Semen parameters	Mean ± SD	Categories	*n* (%)
Volume (ml)	2.42 ± 1.19	Normal	58 (72.5)
		High	4 (5.0)
		Low	18 (22.5)
pH	7.83 ± 0.47	Normal	55 (68.9)
		High	13 (16.3)
		Low	12 (15)
Active motility (%)	23.10 ± 23.46	High	17 (21.2)
		Low	63 (78.8)
Sluggish motility (%)	24.08 ± 16.62	High	32 (40.0)
		Low	48 (60.0)
Immotile (%)	47.68 ± 30.22	High	47 (58.8)
		Low	33 (41.3)
Ideal morphology (%)	1.06 ± 0.24	High	75 (93.8)
		Low	5 (6.3)
Viability (%)	1.60 ± 0.54	High	29 (36.2)
		Low	51 (63.8)
Count (×10^6^/ml)	22.84 ± 21.6	Normospermia	45 (56.3)
		Oligospermia	31 (38.8)
		Azoospermia	4 (5.0)

**Table 3:** Effect of lifestyle on semen parameters in Ghanaian men.

Lifestyle	Semen parameters						
	Volume	pH	Active motility (%)	Sluggish motility (%)	Immotile (%)	Viability (%)	Count (×10^6^/ml)
Alcohol							
Yes	2.23 ± 1.16	7.94 ± 0.41	23.75 ± 20.20	26.60 ± 18.36	42.14 ± 27.66	44.64 ± 25.74	25.00 ± 22.14
No	2.52 ± 1.20	7.77 ± 0.50	22.75 ± 25.22	22.73 ± 15.62	50.67 ± 31.35	38.55 ± 28.23	21.68 ± 21.44
p value	0.295	0.108	0.847	0.323	0.214	0.334	0.516
Smoking							
Yes	2.48 ± 1.10	7.78 ± 0.39	12.85 ± 17.04	22.85 ± 22.14	48.57 ± 34.84	27.85 ± 27.05	12.28 ± 10.95
No	2.41 ± 1.20	7.84 ± 0.48	24.08 ± 23.84	24.20 ± 16.19	47.60 ± 30.01	41.91 ± 27.27	23.85 ± 22.14
p value	0.877	0.741	0.147	0.839	0.936	0.198	0.036^∗^
Hours of sitting							
≤4 hours	2.39 ± 1.26	7.81 ± 0.50	26.02 ± 24.95	24.18 ± 17.14	41.42 ± 30.12	42.24 ± 27.52	23.03 ± 20.75
>4 hours	2.46 ± 1.07	7.87 ± 0.44	18.48 ± 20.42	23.93 ± 16.03	57.58 ± 28.07	38.22 ± 27.43	22.54 ± 23.47
p value	0.791	0.590	0.145	0.948	0.017^∗^	0.526	0.923

∗Statistically significant *p* value < 0.05.

**Table 4:** Effect of exposure to environmental factors on semen parameters in Ghanaian men.

Environmental factors	Semen parameters						
	Volume	pH	Active motility (%)	Sluggish motility (%)	Immotile (%)	Viability (%)	Count (×10^6^/ml)
STD exposure							
Yes	2.32 ± 1.02	7.87 ± 0.45	18.90 ± 21.01	28.10 ± 21.86	42.50 ± 30.97	40.25 ± 26.03	20.22 ± 19.73
No	2.45 ± 1.24	7.82 ± 0.48	24.50 ± 24.22	22.75 ± 14.45	49.41 ± 30.02	40.83 ± 27.90	23.60 ± 22.30
*p* value	0.632	0.662	0.328	0.215	0.379	0.933	0.565
X-ray exposure							
Yes	1.98 ± 0.87	7.88 ± 0.48	34.44 ± 28.77	33.33 ± 23.97	20.00 ± 18.02	58.33 ± 26.80	34.55 ± 24.63
No	2.47 ± 1.21	7.82 ± 0.48	21.66 ± 22.54	22.91 ± 15.29	51.19 ± 29.70	38.45 ± 26.81	21.35 ± 20.92
*p* value	0.158	0.724	0.124	0.076	0.000^∗^	0.039^∗^	0.084
Site of mobile phone storage							
Side pocket	2.41 ± 1.24	7.82 ± 0.48	25.26 ± 24.45	24.95 ± 16.48	46.69 ± 29.73	43.83 ± 27.19	24.16 ± 22.32
Other places	2.45 ± 0.87	7.8 ± 0.43	10.83 ± 10.83	19.16 ± 17.29	53.33 ± 33.66	22.91 ± 21.79	15.33 ± 15.62
*p* value	0.884	0.741	0.002^∗^	0.269	0.486	0.009	0.109

^∗^Statistically significant *p* value < 0.05.

## Data Availability

The authors of this article declare that the data for this article is available, and are willing to share it, when needed.
